# Supplementary material for: Splice-Junction-Based Mapping of Alternative Isoforms in the Human Proteome
Source: Cell Rep. Author manuscript; Available in PMC 2020 Jan 15. (PMC6961840; doi:10.1016/j.celrep.2019.11.026)

A

sp|P53675|CLH2_HUMAN|ENSG00000070371|RI1|88|chr22|19196415|19196656|−2|r12|T4
LQEHFQLQNLGINPANIGFSTLTM[15.99]ESDK q value: 2.9253e−05 Tr_novel:TRUE RefSeq_Novel:TRUE
Search result spec prec mz: 1055.1949 Actual spec prec mz: 1055.1948
Fragments matched per AA: 1.07 Proportion of top 20 peaks matched: 0.4

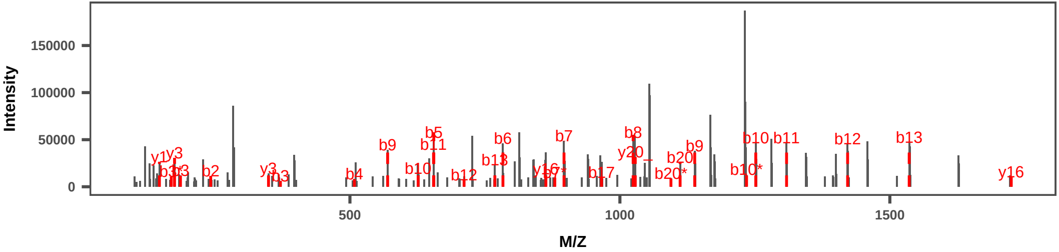

B

Scatterplot of predicted elution time
Fitting R2: 0.865
Novel peptide residual Z score: −1.26
Number of peptides: 2331

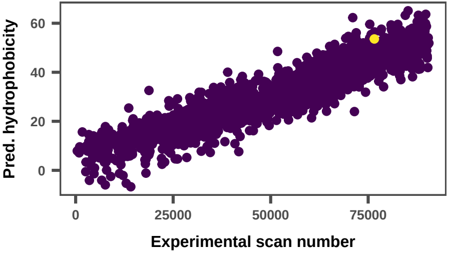

C

Distributions of residuals from best−fit line
of predicted RT vs Expt. scan number
Line: Z score of novel peptide
Z: −1.26

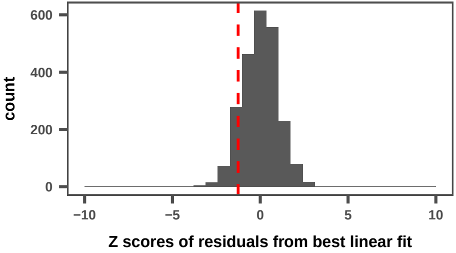

Supplement: 2 [file NIHMS1546469-supplement-2.zip › DF1/PXD006675/RightAtrium/RightAtrium_1_CLTCL1_LQEHFQLQNLGINPANIGFSTLTMESDK.pdf]
